# Behavioral escalation and medical compliance in ASD: is escape extinction really necessary? The effectiveness of the Universal Protocol

**DOI:** 10.1192/j.eurpsy.2026.11166

**Published:** 2026-07-31

**Authors:** M. De Toffol, L. Franco, S. Vergine, A. Rosato, C. Pedaci, M. Vergine

**Affiliations:** Amici di Nico, Lecce, Italy

## Abstract

**Introduction:**

Children with Autism Spectrum Disorder (ASD) often struggle during medical procedures, exhibiting problem behaviors that interfere with care delivery and increase the risk of restraint or sedation. Although traditional behavioral protocols based on graduated exposure and differential reinforcement are effective, they typically include escape extinction, which may lead to emotional escalation and damage the therapeutic relationship. Emerging evidence suggests that alternative protocols—free from extinction and based on precursor reinforcement—may yield comparable or superior outcomes. However, current data are very limited and come from very small samples.

**Objectives:**

To compare the effectiveness of the Universal Protocol (Hanley) with a traditional treatment in promoting medical compliance in children and adolescents with ASD.

**Methods:**

Twenty subjects diagnosed with ASD were assigned to two groups (n = 10 each). The traditional group received differential reinforcement, shaping, stimulus fading, and escape extinction. The Hanley group received the same components, with the addition of precursor reinforcement, increased engagement, escalation prevention, choice opportunities, personalized decision-making and absence of escape extinction. The target behavior involved a 7-step progressive tolerance training culminating in an actual venous blood draw. We collected the number of trials required to pass each step, total trials, and total problem behaviors. Between-group comparisons were conducted using Mann–Whitney U tests.

**Results:**

The Hanley group required significantly fewer total trials (median = 120 vs 220; p < 0.002) and exhibited far fewer problem behaviors (median = 0.5 vs 32.5; p < 0.001). Step-by-step analysis showed that the Hanley group required fewer median trials for steps 2, 3, 4 and 7 and exhibited a complete reduction in problem behaviors across all steps, with statistically significant differences compared to the traditional group.

**Table 1.** Table 1. Long description.

Outcome	Median - Traditional	Median - Hanley	p-value	Cliff’s δ (95% CI)
Total trials	220	120	< 0.002	–0.82 [–1.00 ; –0.46]
Total problem behaviors	32.5	0.5	< 0.001	–0.95 [–1.00 ; –0.79]

**Conclusions:**

The Universal Protocol (Hanley), implemented in naturalistic clinical settings and without escape extinction, proved more effective and less distressing than traditional methods. These results support the adoption of safe, compassionate, and observable interventions, which promote emotional safety, strengthen the therapeutic relationship, and prevent escalation through non-coercive approaches.

**Disclosure of Interest:**

None Declared

## Long descriptions

### Table 1. Long description

The table consists of five columns: Outcome, Median - Traditional, Median - Hanley, p-value, and Cliff’s delta with a 95 percent C I.

* Row 1: Total trials. Traditional median is 220. Hanley median is 120. The p-value is less than 0.002. Cliff’s delta is negative 0.82 with a 95 percent C I of negative 1.00 to negative 0.46.

* Row 2: Total problem behaviors. Traditional median is 32.5. Hanley median is 0.5. The p-value is less than 0.001. Cliff’s delta is negative 0.95 with a 95 percent C I of negative 1.00 to negative 0.79.

Navigate back to Table 1..

